# Chronic nonbacterial osteomyelitis: a typical case and review

**DOI:** 10.3389/fped.2026.1747342

**Published:** 2026-02-27

**Authors:** Qiang Li, Yiwei Wang, Fei Liu, Pengfei Zheng

**Affiliations:** Departmet of Orthopaedic Surgery, Children’s Hospital of Nanjing Medical University, Nanjing, Jiangsu, China

**Keywords:** autoinflammatory diseases, bone inflammation, chronic non-bacterial osteomyelitis, chronic recurrent multifocal osteomyelitis, magnetic resonance imaging (MRI)

## Abstract

**Background:**

Chronic Non-bacterial Osteomyelitis (CNO) is a rare autoinflammatory bone disease primarily affecting children and adolescents. The disease presents with a wide spectrum of severity, ranging from mild unifocal lesions to severe, recurrent multifocal bone inflammation. Its etiology remains unclear, making diagnosis challenging due to nonspecific symptoms.

**Methods:**

We report the case of a 14-year-old girl who presented with recurrent swelling and pain in the left clavicle. After multiple admissions, the patient underwent extensive diagnostic workup, including laboratory tests, imaging, and biopsies, which showcased typical imaging and histopathological findings throughout the disease progression, helping to rule out infections and malignancies. Based on clinical findings and the exclusion of other conditions, she was diagnosed with CNO. Treatment included NSAIDs, intravenous antibiotics, and oral medications such as diclofenac sodium, naproxen, methotrexate, and calcitriol.

**Results:**

During the one-year follow-up after initial treatment, the patient experienced recurrent symptoms, including swelling and pain in the left clavicle. After escalation to intravenous pamidronate and subcutaneous adalimumab, the patient achieved sustained clinical remission. During the subsequent two-year follow-up, no further symptom recurrence was observed.

**Conclusion:**

CNO is generally diagnosed by exclusion, with MRI being the gold standard for detecting asymptomatic lesions and assessing disease activity. Treatment typically involves NSAIDs, with bisphosphonates and biologics increasingly used in refractory cases. This case underscores the complexity of diagnosing and managing CNO, highlighting the need for a multidisciplinary approach. Further research is essential to establish standardized diagnostic criteria and optimize treatment strategies for this rare condition.

## Introduction

Chronic Non-bacterial Osteomyelitis (CNO) is an autoinflammatory bone disease, primarily affecting children and adolescents, with a reported prevalence of 0.4–2.3 in 100,000 and a female-to-male ratio ranging from 2:1 to 4:1 (1–6), first described by Giedion et al., it manifests as subacute or chronic symmetrical osteomyelitis (7). CNO ranges from mild, limited unifocal lesions to severe, chronically active or recurrent multifocal bone inflammation. The latter is the severe form of CNO and is called Chronic Recurrent Multifocal Osteomyelitis (CRMO). CNO is often used to refer to the entire spectrum of this disease (8, 9).

The etiology of CNO remains unclear, and recent studies continue to report potential new pathogenic genes (10). Given its variable presentation, ranging from mild unifocal lesions to severe multifocal inflammation, CNO poses significant diagnostic challenges, particularly due to its nonspecific symptoms and the lack of a clear diagnostic biomarker. It is often made by a process of exclusion. Clinical assessment includes auxiliary examinations such as serum inflammatory markers and imaging tests, especially MRI (11). Biopsy is often required to exclude infectious osteomyelitis or malignant bone tumors (12). In terms of treatment, Nonsteroidal Anti-Inflammatory Drugs (NSAIDs) remain effective for most patients, while pamidronate is particularly effective for patients with spinal involvement and structural damage (13–15) For the very few patients who do not respond to the above treatments, other biological agents may be considered (16, 17). Despite emerging research, much remains unknown about CNO, particularly in the context of its pathogenesis and optimal treatment strategies.

Here, we report a real-world case of unifocal clavicular CNO with a complicated disease course, including repeated admissions, prolonged antibiotic therapy, and partial clavicle resection before the correct diagnosis was established. Subsequently, the case was successfully diagnosed through interdisciplinary collaboration among the departments of Orthopedics, Rheumatology and Immunology, and Radiology, with clinical improvement following treatment. Building on this case, we also provide an updated literature review to improve clinical awareness and diagnostic reasoning in children presenting with CNO.

## Method

### First admission

**Figure 1:**
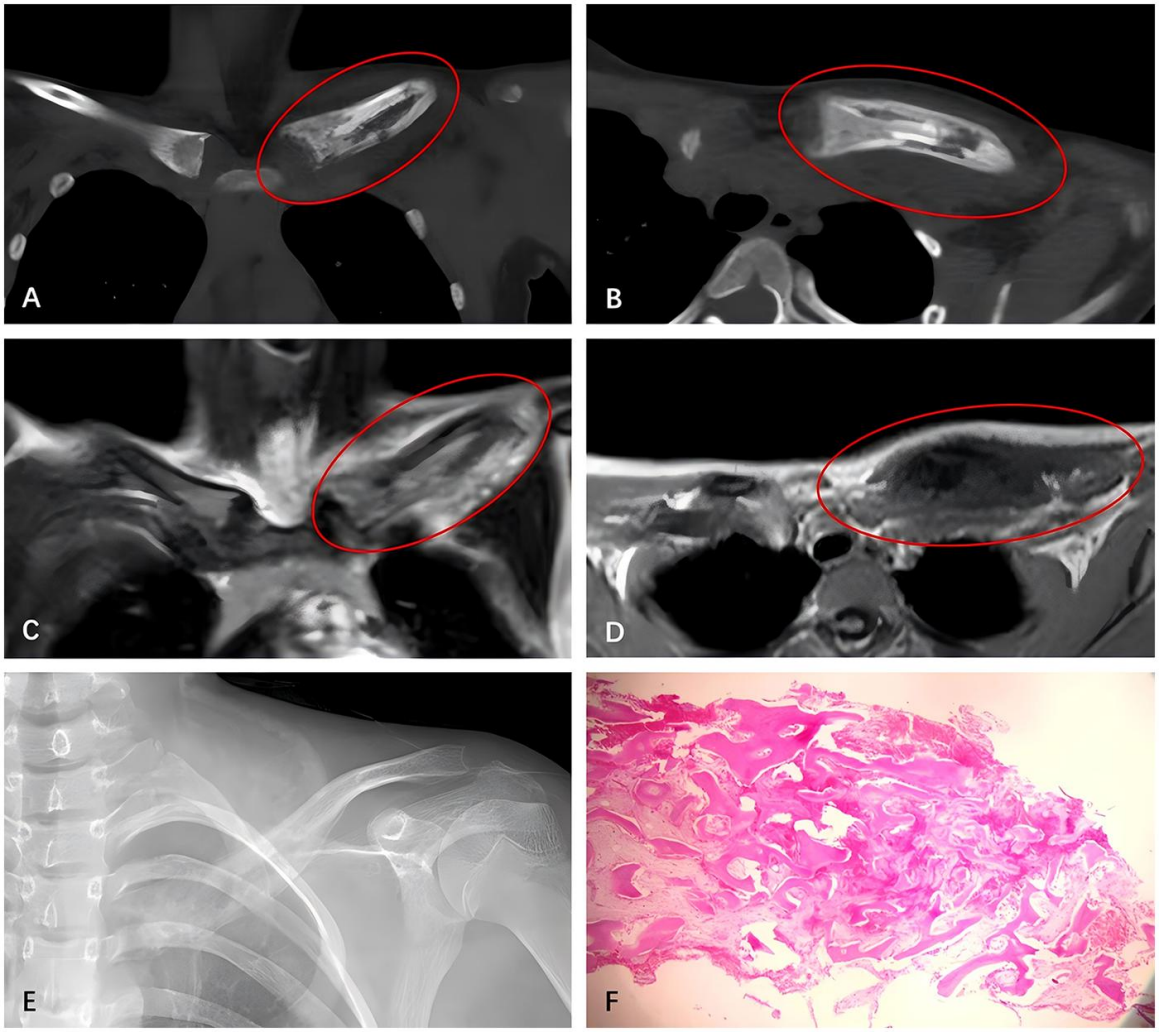
CT **(A,B)** and MRI **(C,D)** images of the left clavicle at the initial admission: enlargement of the left clavicle with uneven bone density and evidence of moth-eaten bone destruction. The cortical bone is discontinuous, with some periosteal reaction observed. Postoperative x-ray images following the first surgery **(E)**: the proximal left clavicle has been removed. Slight enlargement of the left clavicle near the sternoclavicular joint, with uneven bone density. Histological examination **(F)**: proliferative sclerotic bone, fibrous tissue proliferation between bone trabeculae, vascular dilation, and significant chronic inflammatory cell infiltration, with focal plasma cell clusters.

In June 2021, the 14-year-old girl presented with swelling and pain in the left clavicle. Laboratory tests showed elevated C-Reactive Protein (CRP) (11 mg/L) and a White Blood Cell (WBC) count of 6.5 × 10⁹/L, while blood cultures were negative. CT and MRI revealed osteolytic changes and periosteal reaction in the left clavicle (Figures 1A–D). Two months later, due to lack of improvement, the patient underwent partial resection and biopsy of the clavicle lesion, which showed chronic inflammatory cell infiltration without signs of malignancy (Figures 1E,F). The patient was treated with intravenous antibiotics, and her symptoms improved.

### Second admission

**Figure 2:**
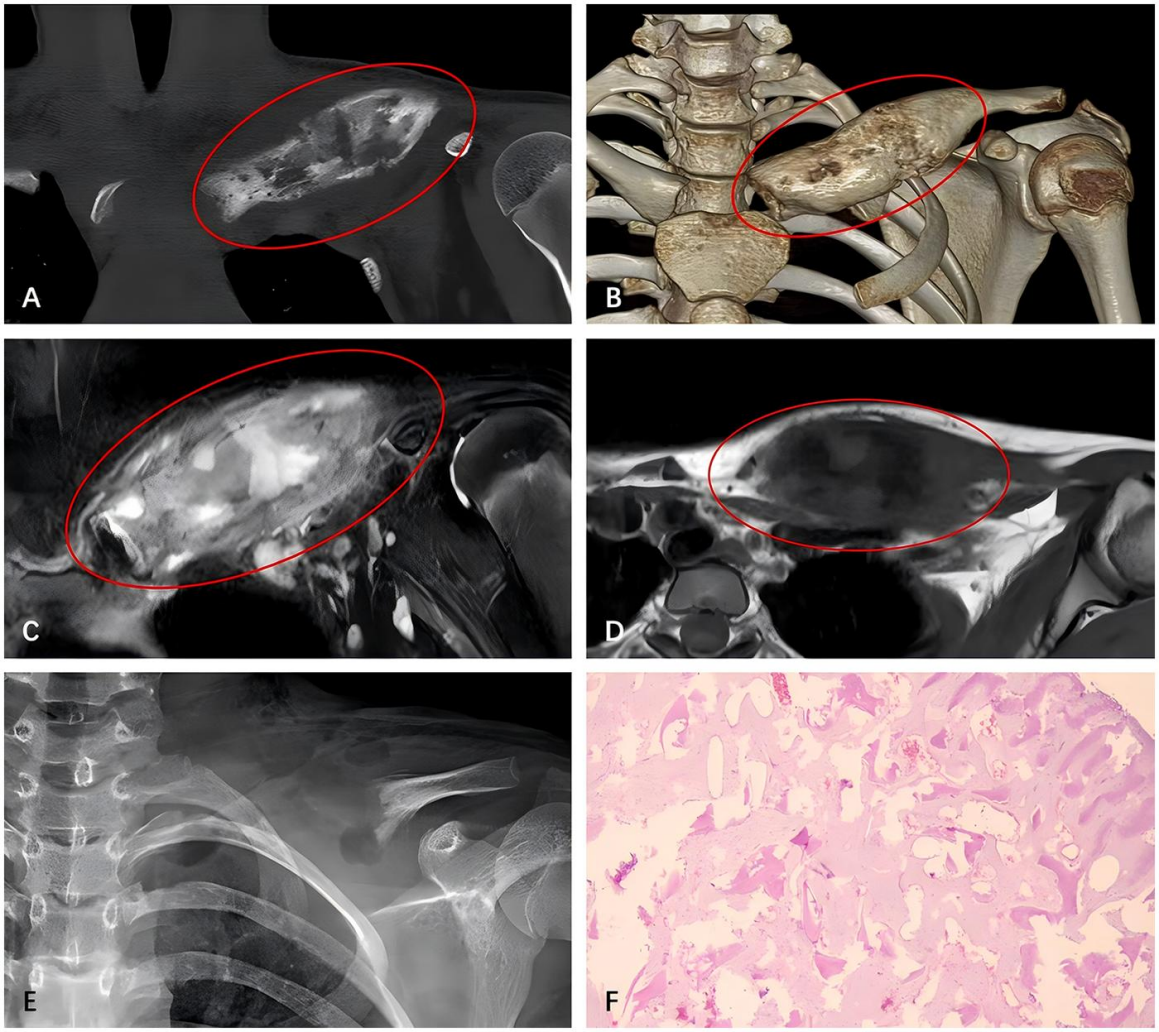
CT **(A,B)** and MRI **(C,D)** from the second admission: the lesion at the sternoclavicular end of the clavicle has become more enlarged and thickened compared to previous images, showing similar osteolytic changes and periosteal reaction. Postoperative x-ray images following the second surgery **(E)**: the proximal bone of the left clavicle missing, with uniform bone density at the distal end. Histological examination **(F)**: proliferative fibrous tissue and irregular bone trabeculae. Active osteoblast coverage is observed around the bone trabeculae, with focal tissue reactions and giant cell proliferation. Plasma cell infiltration is seen in the surrounding stroma, and reactive bone formation is present.

In January 2022, the patient again presented with swelling and pain in the left clavicle. Laboratory tests showed elevated CRP (27.94 mg/L) and a WBC count of 6.99 × 10⁹/L. Blood cultures remained negative, and CT and MRI results were similar to the previous imaging (Figures 2A–D). The patient was treated with intravenous vancomycin. A second excisional biopsy of the left clavicle was performed, and although the possibility of Paget's disease and Fibrous Dysplasia (FD) was considered, the etiological examination remained negative (Figures 2E,F). Based on her short-term history of antibiotic use and previous medical history, she was still diagnosed with chronic osteomyelitis of the left clavicle. During treatment, the patient developed a pustule on her right heel. Histopathological examination ruled out fungal infection, and her condition improved after applying etofesalamide ointment. The patient's clavicle symptoms also improved after the antibiotic therapy, with no recurrence during subsequent outpatient follow-ups.

### Third admission

**Figure 3:**
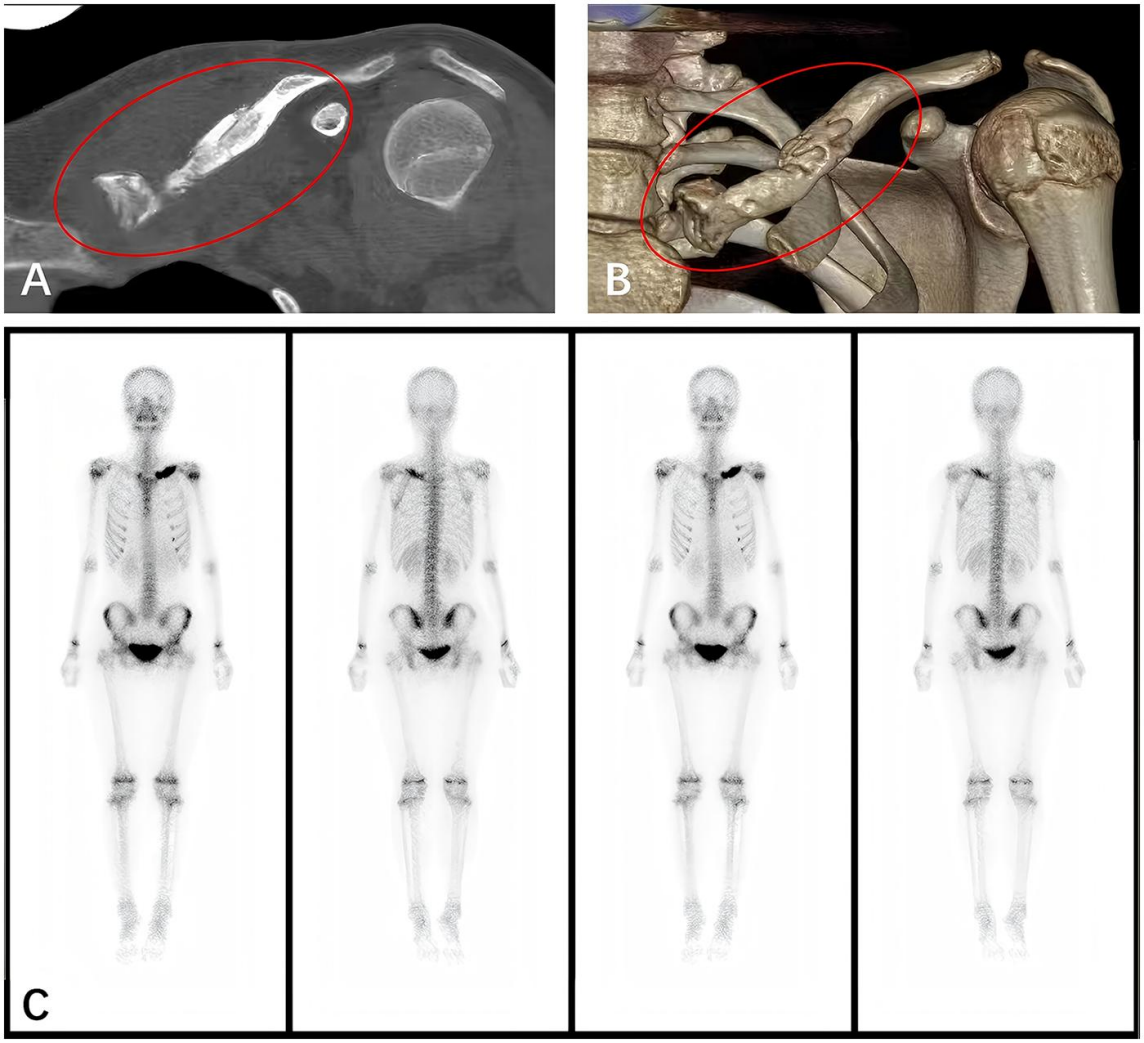
CT images **(A,B)** from the third admission: the left clavicle is slightly shortened, with local bone enlargement, uneven bone density, and a slightly irregular contour. Mild periosteal reaction is observed. Whole-body bone scan **(C)** showing increased uptake in the left clavicle, with no other skeletal involvement.

In February 2023, the patient presented again with swelling and pain in the left clavicle. Laboratory tests showed elevated CRP (20.11 mg/L) and a WBC count of 9.19 × 10⁹/L. CT results were similar to previous findings (Figures 3A,B). The patient was treated with intravenous cephalosporins, but her symptoms showed no significant improvement. Consequently, the orthopedic department led a comprehensive differential diagnosis in collaboration with rheumatology and radiology specialists. Bacterial osteomyelitis was initially considered but was subsequently excluded based on repeatedly negative blood cultures, the absence of identified pathogens, and the lack of sustained clinical response to prolonged antibiotic therapy. Two separate bone biopsies both revealed chronic inflammatory changes, no evidence of bacteria or tumor cells. Due to the absence of characteristic histopathological features, Langerhans cell histiocytosis, fibrous dysplasia, and Paget's disease were excluded. Metabolic bone disorders, including hypophosphatasia, were also considered but were excluded based on normal biochemical parameters. At this point, considering the patient's medical history and the absence of signs indicating spinal or other skeletal involvement, a diagnosis of “unifocal clavicular CNO” was considered. Although the gold standard for diagnosing CNO is whole-body MRI, the parents declined this examination due to financial constraints following discussions with the medical team. Tc-99m-MDP bone scintigraphy was therefore performed. The results revealed increased inorganic salt metabolism limited to the left clavicle, with no evidence of additional skeletal lesions, which is consistent with the diagnosis of unifocal CNO (Figure 3C).

Based on the Jansson criteria (18), the patient fulfilled two major criteria—typical imaging findings consistent with CNO and chronic inflammatory histopathology—and three minor criteria, including mildly elevated CRP levels, normal blood counts, and absence of systemic infection. Although the disease presentation was unifocal, the overall clinical and pathological findings met the diagnostic threshold defined by the Jansson criteria, thereby supporting the diagnosis of CNO. Subsequently, the patient was transferred to the rheumatology and immunology department for oral medications, which included diclofenac sodium, naproxen, methotrexate, calcitriol and total glucosides of paeony (19) (a modern herbal medicine preparation extracted from the dried root of Paeonia lactiflora Pallas).

## Results

Following the initiation of oral anti-inflammatory and immunomodulatory therapy, the patient experienced gradual improvement in pain and swelling of the left clavicle.

During the subsequent follow-up period, intermittent mild symptoms persisted. Given the recurrent nature of symptoms and incomplete clinical remission, treatment was escalated to intravenous pamidronate and subcutaneous adalimumab. Pamidronate was applied at 1 mg/kg monthly (45 mg), which was repeated for half a year.

**Figure 4:**
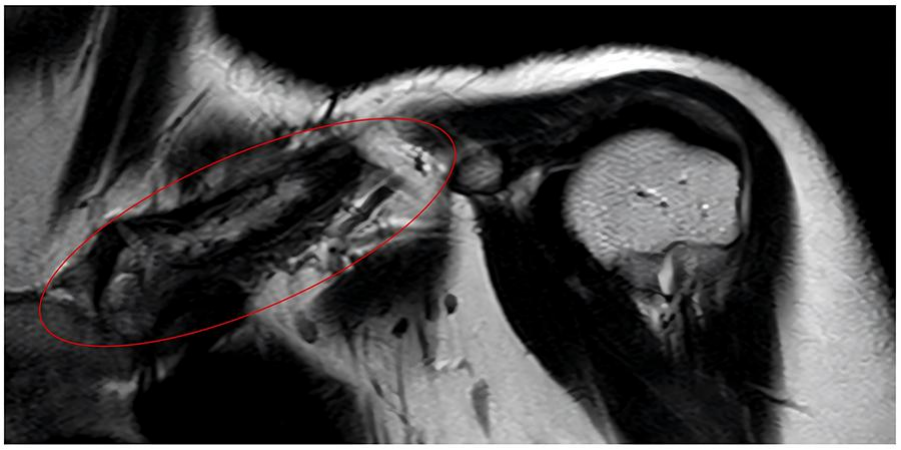
Follow-up MRI: persistent structural changes, including osteolytic destruction and periosteal reaction, are observed in the left clavicle.

After treatment escalation, the patient achieved sustained clinical remission, with complete resolution of clavicular pain and swelling and normalization of inflammatory markers. Follow-up MRI demonstrated persistent structural changes of the left clavicle with periosteal reaction, but no progression of lesions and no newly detected bone involvement (Figure 4).

At the most recent follow-up, approximately 3 years after the third admission, the patient remained clinically stable without recurrence of symptoms. No spinal involvement, neurological symptoms, or functional limitations were observed during long-term follow-up.

## Discussion

The present report illustrates a protracted and diagnostically challenging case of unifocal clavicular CNO in an adolescent girl. While pediatric CNO is a well-described entity in the rheumatology literature, its recognition and management remain problematic in routine clinical settings, particularly when it presents atypically. This case not only reinforces established challenges but also provides several crucial, practice-oriented insights。.

The significance of this case extends beyond its rarity. First, unlike almost all previously published reports, this case is presented from the perspective of orthopedic surgeons rather than rheumatologists, providing practical experience in the diagnosis and management of CNO from an orthopedic standpoint. At the same time, the case reflects the real-world early diagnostic and treatment pathway of a CNO patient, complementing the rheumatologists' understanding of pre-referral clinical course. Second, this case highlights the tortuous medical journey of this CNO patient, who suffered prolonged misdiagnosis and multiple unnecessary surgeries. It can help readers of this article to be vigilant and make thorough differential diagnoses when encountering similar problems, and to avoid these diagnostic pitfalls as much as possible. Third, the diagnostic and therapeutic journey of this case provide valuable insights from both positive and negative perspectives. On one hand, in the current absence of standardized protocols for CNO, the effective therapeutic approach explored in this case offers practical reference for its clinical management. On the other hand, the prolonged misdiagnosis and multiple inappropriate treatments prior to definitive diagnosis fully reveal the complexity of diagnosing and managing CNO, serving as a critical reminder for orthopedic surgeons when encountering such challenging cases. Finally, the successful diagnosis and management of this patient ultimately depended on close interdisciplinary collaboration among orthopedics, radiology, and rheumatology. This underscores that, not only for CNO but also for other diagnostically challenging conditions, early multidisciplinary involvement may substantially improve diagnostic accuracy and clinical outcomes.

### Pathogenesis

The exact pathogenic mechanism of CNO is still unclear. Studies by Girschick et al. have shown that no pathogenic bacteria were detected in lesions using PCR and other techniques, and antibiotic treatment was ineffective, confirming that CNO is a sterile inflammatory disease (20). Research by Hofmann et al. has demonstrated that chronic inflammation in CNO patients may be driven by the pro-inflammatory phenotype of monocytes. These studies revealed elevated levels of inflammatory cytokines, such as interleukin-1β (IL-1β), IL-6, and Tumor Necrosis Factor-α (TNF-α), while the expression of regulatory cytokines IL-10 and IL-19 was reduced (21–23) Although these findings suggest a link between cytokine imbalance and disease progression, further research is needed to clarify this relationship and to explore targeted immunotherapies.

Genetic abnormalities have also been implicated in CNO pathogenesis. For example, Majeed syndrome, which is caused by homozygous mutations in the LPIN2 gene, is characterized by recurrent fever, CNO, and Congenital Dyserythropoietic Anemia (CDA) (24). Another condition associated with CNO is Deficiency of Interleukin-1 Receptor Antagonist (DIRA), caused by mutations in the IL1RN gene. This mutation leads to unchecked activation of the interleukin-1 receptors, triggering CNO symptoms (25). Cox et al. (26) suggested that the FBLIM1 gene, which encodes filamin-binding LIM protein 1, may also be involved in CNO pathogenesis. A cohort study by d'Adamo et al. identified rare high-frequency variants of FBLIM1 in the Italian population (27). Açarı et al. reported MEFV gene mutations in several patients (4), while Zhao et al's (10) study on 10 patients revealed mutations in COL1A1, PSTPIP1, LRP5, and CLCN7. Important recent studies, such as Charras et al., have identified P2RX7 gene variants linked to altered inflammasome activity and cytokine release in CNO (28). The c.349C > T/p.Arg117Trp variant was found to enhance NLRP3 inflammasome assembly, highlighting a genetic factor in CNO's pathogenesis and offering potential for targeted therapies. Despite these findings, the role of genetics in CNO's pathogenesis requires further exploration.

### Clinical presentation

The clinical manifestations of CNO can vary significantly among patients. Most children experienced localized bone pain, often accompanied by tenderness and swelling. However, some lesions are asymptomatic. Almost all bones can be affected but the metaphysis of long bones is most commonly affected in children. The femur, tibia and vertebrae are the most commonly affected sites, with the clavicle and mandible frequently involved in unifocal cases (29–31) Recent research indicates that the pelvis and sacroiliac joint are also typical sites of involvement and should be considered key areas of focus in disease assessment (32). Up to one-third of patients develop vertebral inflammation, which can progress to vertebral compression fractures or kyphosis (18, 31). 17%–20% of children may experience mild systemic symptoms such as fever, fatigue, and weight loss, but these are usually mild and not persistent (33, 34). A cohort study by Borzutzky A et al. (33) showed that half of children with CNO had at least one autoimmune disease, and 49% of them had a family history of autoimmune diseases, including psoriasis, palmoplantar pustulosis, acne, inflammatory arthritis, or inflammatory bowel disease (29, 35–37). These features significantly overlap with the clinical presentation of SAPHO syndrome (synovitis, acne, pustulosis, osteophyte formation, osteitis), suggesting that they belong to the same disease spectrum.

Compared to pediatric patients, adults were more frequently diagnosed with skin lesions (41% vs. 19%), with palmoplantar pustulosis (PPP) being the most common presentation in adults, while severe acne was the most common presentation in children (38). Furthermore, the sternum and vertebrae were the most common sites of involvement in adult patients (39, 40). SAPHO is more common in adults and may represent a manifestation of CNO in this population, while pediatric patients are more likely to present with core skeletal inflammation of CNO/CRMO (29, 41, 42).

Cebecauerová et al. classified CNO/CRMO into two categories based on the presence or absence of extraosseous manifestations, highlighting the need for further investigation into the connection between these two conditions (43). In this case, the patient had a history of right heel pustules, which were initially overlooked. This emphasizes the need to consider SAPHO syndrome (synovitis, acne, pustulosis, hyperostosis, osteitis) during the diagnostic process to avoid misdiagnosis and delayed treatment.

### Imaging findings

Imaging studies often begin with the symptomatic site. Common imaging modalities include x-rays, CT, MRI, and whole-body diffusion-weighted imaging (WB-DWI) (44). While x-rays are useful for differentiating fractures, early-stage CNO lesions are typically negative on x-rays, necessitating more sensitive imaging methods (44). CT scans provide clearer structural details, but their use should be minimized due to radiation exposure, especially in children (45).

MRI is the first choice for diagnosing CNO due to its superior sensitivity compared to x-rays and CT (46). Typical MRI findings in CNO include bone marrow hyperintensity on STIR sequences, soft tissue/periosteal hyperintensity, and bony expansion (47). Early CNO lesions appear as geographic periphyseal bone marrow oedema on fluid-sensitive sequences like STIR. As oedema extent progresses over time, chronic CNO lesions develop irregular outlines. Corresponding T1 hypointensity varies and some preserved T1 fatty marrow signal can remain. Dense sclerosis in CNO lesions can manifest as STIR and T1 hypointensity that mimics marrow infiltration or tumour new bone formation (48). Whole-body MRI is the gold standard for diagnosing CNO, capable of identifying clinically asymptomatic lesions and assessing disease activity (49). Guariento et al. reported that the thoracic spine is the most frequently affected area in CNO patients, with kyphosis or scoliosis present in one-quarter of cases and reduced vertebral height in one-third (50). Kaut et al. confirmed MRI-detected lesions in 131 out of 180 symptomatic sites in a multicenter study of 30 children (2). Although MRI plays a crucial role in CNO diagnosis, its findings can sometimes be nonspecific. Consequently, imaging results should always be interpreted in conjunction with clinical evaluations and other diagnostic tests to confirm CNO and rule out other conditions (51). Due to the rarity and atypical imaging features of CNO, there is a risk of misdiagnosis. Forestieri et al. explored the application of machine learning algorithms to whole-body MRI to assist in CNO diagnosis, offering a potential method to enhance diagnostic accuracy in the future (52). WB-DWI is used in some centers, but its evidence is limited to small case series. Leclair et al. demonstrated that WB-DWI could reliably detect lesions in children with CNO, providing higher ADC values in lesions compared to reference regions, which may help distinguish benign inflammatory processes from malignancies (53).

In this case, the patient underwent early MRI of the left clavicle, followed by Tc-99m-MDP bone scintigraphy. While whole-body MRI was recommended for better sensitivity, it was not performed as the patient's family declined due to financial constraints, which further highlights the challenges of diagnosing CNO in routine clinical practice, as access to advanced imaging modalities may be limited in such cases.

### Bone biopsy

The role of bone biopsy in diagnosing CNO remains controversial due to the nonspecific nature of its histopathological findings. CNO typically begins with acute inflammation characterized by polymorphonuclear leukocytosis and bone resorption (1). While features such as the coexistence of “acute” inflammatory infiltrates (neutrophils, macrophages) alongside chronic inflammation (characterized by lymphocytes, plasma cells, and monocytes), and/or bone sclerosis are more common in CNO than in other conditions, these findings are not specific and may also be observed in chronic infections, complicating diagnosis (20, 54–56) While biopsy may not offer a definitive diagnosis for CNO, it remains essential for differentiating between CNO and more serious conditions, such as malignancies or chronic infections (55).

In this case, two biopsies were performed to rule out infection and malignancy. Both showed chronic inflammatory infiltration without evidence of bacteria or tumor cells, supporting the diagnosis of chronic nonbacterial osteomyelitis.

### Diagnosis and differential diagnosis

Currently, there is no gold standard for diagnosing CNO, and it remains a diagnosis of exclusion based on clinical history, imaging, laboratory findings, and histopathology. Diagnostic criteria proposed by Jansson et al. in 2007 are commonly used, requiring either two major criteria or one major criterion plus two minor criteria for diagnosis (18). Major criteria include the presence of at least two bone lesions, typical imaging findings such as osteolytic or sclerotic lesions, and histopathological signs of chronic inflammation. Minor criteria involve mild to moderate increases in inflammatory markers like CRP and Erythrocyte Sedimentation Rate (ESR), the absence of systemic symptoms, and a normal complete blood count.

Given the rarity of CNO, accurate diagnosis is often delayed. Previous studies have shown that it can take 1–2 years and multiple biopsies to confirm the diagnosis (45, 57). Leerling et al. reported that the diagnosis can be delayed by as much as five years (58). Since the symptoms of CNO overlap with those of bone infections, many patients initially seek treatment in orthopedic departments. However, because CNO is a systemic autoinflammatory condition, early collaboration between orthopedic and rheumatology or immunology departments is crucial to avoid misdiagnosis or delayed diagnosis.

### Treatment

The management of Chronic Nonbacterial Osteomyelitis (CNO) currently lacks unified, high-level evidence-based guidelines and is primarily based on expert consensus, retrospective studies, and clinical experience (14, 44, 59). The goals of therapy are to reduce inflammation, controll symptoms, induce remission, and minimize long-term complications. The following section systematically outlines current therapeutic strategies, drug options, response assessment, and future directions.

Current Landscape and Principles:

The treatment of CNO remains largely empiric, as no drug is formally approved by regulatory agencies for this condition, making all therapies off-label. Recently, the development of international Consensus Treatment Plans (CTPs) aims to standardize clinical practice, reduce variability in management approaches, and lay the groundwork for future comparative effectiveness studies (60). Therapeutic approaches generally follow a “step-up” strategy: starting with first-line agents and escalating to second- or third-line treatments in cases of insufficient response or high-risk features (e.g., spinal involvement).

Current Therapeutic Strategies:

First-Line Treatment: Nonsteroidal Anti-Inflammatory Drugs

Nonsteroidal anti-inflammatory drugs (NSAIDs) are the first-line choice for patients without spinal involvement or structural damage (61, 62). Their mechanisms of action include inhibition of cyclooxygenase, reduction of prostaglandin synthesis (thereby providing analgesia and inhibiting osteoclast activity), and potential modulation of inflammasome activity (63). Studies indicate that approximately 50%–60% of patients achieve clinical remission within 3–6 months of treatment (61). However, long-term follow-up reveals a high relapse rate, with about 50% of patients experiencing symptom recurrence within 2 years (64). Consequently, a majority of patients eventually require more potent therapies.

2. Second-Line and Subsequent Therapies

For patients with an inadequate response to NSAIDs, spinal lesions (particularly with a risk of vertebral fracture), or high disease activity, second-line agents are added or substituted. The CTPs suggest three main second-line options (60).

Among Conventional disease-modifying antirheumatic drugs (DMARDs), Methotrexate and Sulfasalazine are used for their effect in anti-inflammatory. A few retrospective studies have shown significant variability in their efficacy in treating CNO (65–67) In a prospective cohort study by Hofmann et al., sulfasalazine was introduced for patients who did not achieve sufficient improvement with NSAIDs alone (68). The study showed that sulfasalazine can be an effective part of the treatment strategy in CNO, especially for patients with persistent symptoms or severe disease, but it may not be sufficient for achieving complete remission, particularly when there are significant MRI-detected lesions. The treatment goal for such patients may require further escalation, including the use of biologic therapies or bisphosphonates (68).

Tumor Necrosis Factor-α Inhibitors: Multiple retrospective studies confirm the effectiveness of TNF-α inhibitors (e.g., adalimumab, etanercept, infliximab) in refractory CNO, particularly in cases with associated arthritis, inflammatory bowel disease, or spondylitis (69, 70). Approximately 50%–90.9% of patients achieve clinical and radiological improvement, with effects that may be sustained (40, 71). Kostik et al. reported that TNF inhibitors improved vertebral CNO lesions after pamidronate treatment failed, achieving remission in 38 of 53 children (73%) (71). Notably, TNF-α inhibitor use can induce or exacerbate psoriasis (paradoxical psoriasis) (72, 73).

Bisphosphonates: Intravenous pamidronate is a highly effective treatment for CNO, especially in cases with spinal involvement and structural damage (74). Its mechanism involves inhibition of osteoclast-mediated bone resorption and possible downregulation of pro-inflammatory cytokines (13, 75). Pamidronate has been shown to provide pain relief and promote healing of bone lesions (76, 77). In recent years, the first randomized, double-blind, placebo-controlled preliminary trial conducted by Andreasen et al. further suggests that pamidronate may improve radiological and clinical disease activity in CNO. Zoledronate has been increasingly used in the treatment of CNO. Zhao et al. reported (78) that zoledronate combined with the TNF inhibitor infliximab can achieve rapid onset of action. A series of studies in adult patients showed that zoledronate combined with a TNF inhibitor can effectively relieve pain and local swelling, but further research is needed to confirm the specific effects of the drugs (79–81) Because bisphosphonates may have side effects and have a long biological half-life, they are only used in patients who have not responded to other treatments or have primary vertebral involvement (especially with structural damage) (63).

3. Corticosteroids and Third-Line Biologics

Oral corticosteroids (e.g., prednisolone) are sometimes used as short-term therapy due to their rapid anti-inflammatory effects during acute flares, or be used as bridging therapy during the establishment of DMARD treatment (44). However, long-term use is limited by significant side effects and a high relapse rate after discontinuation, making them unsuitable as maintenance therapy (44, 63, 64).

For the rare patient refractory to the above treatments, other biologic agents may be considered, such as: IL-1 inhibitors (anakinra, canakinumab): May be suitable for patients with monogenic disorders involving the IL-1 pathway (e.g., DIRA, Majeed syndrome) or a subset of sporadic CNO with elevated IL-1β (82–85); IL-6 receptor inhibitor (tocilizumab): Case reports show efficacy in some patients, but data are limited and inconsistent (2, 86, 87).JAK inhibitors (tofacitinib): Show promise in SAPHO syndrome and a few CNO cases, but pediatric experience is minimal, requiring further evaluation of efficacy and safety (88, 89). IL-17 inhibitors (secukinumab) have been primarily studied in adult SAPHO cases and case reports, with limited data in pediatric CNO and variable response rates (90).

The CNO subgroup of the Childhood Arthritis and Rheumatology Research Alliance (CARRA) has developed consensus treatment plans for patients who do not respond to NSAIDs or who present with active spinal lesions. The three consensus plans include: (1) methotrexate or sulfasalazine; (2) TNF-α inhibitors with optional methotrexate; and (3) bisphosphonates (60). These regimens also allow for short courses of glucocorticoids and continued NSAID use to manage acute flares.

Surgical interventions are not recommended for CNO, as the disease is systemic with multifocal involvement and not pathophysiologically suited for surgical intervention.

### Disease activity testing

Monitoring disease activity is crucial in the management of CNO, as many patients experience symptom flares and disease progression over time. However, there are currently no international consensus guidelines specifically for monitoring CNO disease activity. Despite this, several assessment tools have been used in clinical practice to evaluate patient outcomes and manage the disease.

The Pediatric CNO (PedCNO) response score has been developed to assess disease activity by combining several core variables: erythrocyte sedimentation rate (ESR), MRI-defined lesions, physician global assessment of disease activity (PGDA), patient global assessment of disease activity (PAG), and the Childhood Health Assessment Questionnaire (C-HAQ). The score evaluates relative improvements over time, with categories for 30%, 50%, 70%, and 90% improvement. This composite score has been effective in evaluating treatment responses but is somewhat complex to calculate and may not always be practical in routine clinical practice (91, 92).

CNO Clinical Disease Activity Score (CDAS) is the sum of three key variables: patient pain, patient global assessment, and clinical CNO lesion count, used to assess the patient's status over time, focusing on patient-reported pain, global disease activity, and clinical lesions (93). The CNO CDAS has been validated in the CHOIR registry and demonstrated strong correlations with patient/parent global assessment and physician evaluations of disease severity, which demonstrates the potential of this score as a disease severity assessment tool in future research, serving as a feasible and reliable clinical-based assessment tool for disease activity that can be calculated without imaging data (93).

Although these scoring systems provide valuable insights into CNO disease activity, more research is needed to develop a widely accepted, disease-specific tool for monitoring. Such a tool would enable clinicians to make more accurate treatment adjustments based on real-time disease progression and patient symptoms.

## Conclusion

CNO is an autoinflammatory disease closely associated with cytokine imbalances, yet much about its pathogenesis, diagnosis, and treatment remains unknown. The lack of unified diagnostic criteria and treatment guidelines contributes to frequent misdiagnoses and delays in treatment, prolonging the course of the disease and leading to recurrence.

This case report presents the typical imaging and histopathological findings throughout the disease progression of a pediatric CNO patient, providing insights into the challenges of diagnosing and managing this complex condition. The case emphasizes the importance of a multidisciplinary approach, involving close collaboration between orthopedic surgeons, radiologists and rheumatologists for accurate diagnosis and treatment.

Continued research, especially large-sample studies, is essential to deepen our understanding of CNO, its genetic basis, and the effectiveness of emerging therapies. By advancing our knowledge, we can move closer to providing more effective, individualized care for patients suffering from this rare and complex disease.
